# Biaxial Angular Acceleration Sensor with Rotational-Symmetric Spiral Channels and MEMS Piezoresistive Cantilevers

**DOI:** 10.3390/mi12050507

**Published:** 2021-04-30

**Authors:** Rihachiro Nakashima, Hidetoshi Takahashi

**Affiliations:** Department of Mechanical Engineering, Faculty of Science and Technology, Keio University, 3-14-1 Hiyoshi, Kouhoku-ku, Yokohama, Kanagawa 223-8522, Japan; na8.3436@keio.jp

**Keywords:** angular acceleration sensor, piezoresistive cantilever, spiral channel, differential pressure sensor

## Abstract

Angular acceleration sensors are attracting attention as sensors for monitoring rotational vibration. Many angular acceleration sensors have been developed; however, multiaxis measurement is still in a challenging stage. In this study, we propose a biaxial angular acceleration sensor with two uniaxial sensor units arranged orthogonally. The sensor units consist of two rotational-symmetric spiral channels and microelectromechanical system (MEMS) piezoresistive cantilevers. The cantilever is placed to interrupt the flow at the junctions of parallelly aligned spirals in each channel. When two cantilevers are used as the resistance of the bridge circuit in the two-gauge method, the rotational-symmetric spiral channels enhance the sensitivity in the target axis, while the nontarget axis sensitivities are canceled. The fabricated device responds with approximately constant sensitivity from 1 to 15 Hz, with a value of 3.86 × 10^−5^/(rad/s^2^), which is equal to the theoretical value. The nontarget axis sensitivity is approximately 1/400 of the target axis sensitivity. In addition, we demonstrate that each unit responds according to the tilt angle when the device is tilted along the two corresponding rotational axis planes. Thus, it is concluded that the developed device realizes biaxial angular acceleration measurement with low crosstalk.

## 1. Introduction

Angular motion is one of the most significant parameters for controlling and navigating the attitude of a mobile object [1,2,3]. In many cases, absolute angles are desired and calculated by integrating a signal of gyroscopes for which the detection target is angular velocity. However, angular acceleration measurements are essential in some situations, for example, aerial vehicle attitude control systems, inertial navigation, seismic records, and nanoattitude control systems for atomic force microscopes [4,5,6]. Gyroscopes can obtain angular acceleration by differentiating the measured angular velocity signal; however, there is a possibility of missing a tiny rotational vibration through the differentiation process. Thus, direct angular acceleration measurement is suitable for such situations [7]. In addition, a multiaxial angular acceleration component must be measured because motion is three-dimensional in general.

Against this background, several angular acceleration sensors have been developed [8,9,10,11,12,13,14,15,16,17,18,19,20]. For example, some angular acceleration sensors use an individual solid mass structure as the sensor element as well as linear acceleration sensors [8,9,10,11,12]. Other angular acceleration sensors utilize the inertial forces acting on fluid in a circular channel as the detection target [13,14,15,16,17,18,19,20]. The use of fluid inertial force has advantages such as a simple sensor structure and low power consumption. If the ring channel is a completely circular shape, the sensor does not respond at all to angular acceleration around any axis other than the target ring axis. However, the ring channel cannot be perfectly circular due to design considerations, such as vertically shifting the semicircle to place the sensor elements inside the ring, and the sensor responds to the nontarget axial angular acceleration. An angular acceleration sensor using mirror-symmetric ring channels was studied in an attempt to cancel these nontarget sensitivities [21]. On the other hand, sensors using fluid in circular channels have a principle-related weak point; the sensitivity of the device decreases as the size decreases. Thus, it has been difficult to achieve both a compact size and high sensitivity for a ring channel angular acceleration sensor. To solve this problem, a spiral channel was utilized instead of a simple ring channel to amplify the inertial force and improve the sensor sensitivity [22]. Through the improvement in the target axis sensitivity, it was possible to reduce the relative sensitivity of the other axes. Therefore, if a mirror-symmetric structure is applied to a spiral channel, a uniaxial angular acceleration sensor can be realized with significantly high independence in principle. This sensor structure can be utilized for the sensor unit of a multiaxial angular acceleration sensor. However, due to the potential structural complexity, sensors that extend to multiple axes have not yet been developed.

Here, we propose an angular acceleration sensor with spiral channels that achieves biaxial detection with low crosstalk. Given that rotational-symmetric Archimedes’ spiral channels are formed in the same plane, we realize both sensitivity enhancement due to the number of turns in the spiral and the canceling of other axial angular accelerations. As the sensor element, highly sensitive microelectromechanical system (MEMS) pressure sensors are utilized so that the sensitivity is still sufficient for principle verification even if the fluid inside the channel is air. With the orthogonal arrangement of two sensor units using rotational-symmetric channels, biaxial angular acceleration components are detectable.

In the following sections, we introduce the design and theory of the proposed sensor structure. Then, we evaluate the characteristics of the fabricated sensor with regard to the three axial angular accelerations. Finally, we demonstrate the sensor response when the sensor is tilted at a certain angle to the rotating surface.

## 2. Measurement Principle

**Figure 1:**
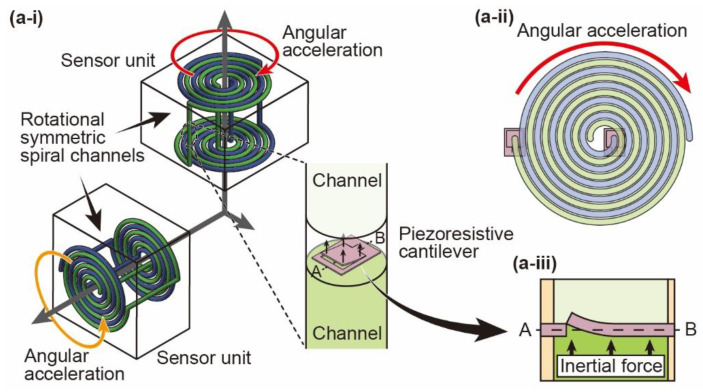
Proposed sensor configuration. (**a-i**) Schematic image of the proposed sensor and enlarged view of the channel connection, (**a-ii**) top-view schematic image of the rotational-symmetric spiral channels, and (**a-iii**) cross-sectional view of the piezoresistive cantilever deformation due to inertial force.

Figure 1 from (a-i) shows the configuration of the proposed angular acceleration sensor. The sensor consists of two orthogonal uniaxial sensor units so that angular acceleration in two axes is detectable. Each sensor unit consists of two independent rotational-symmetric spiral channels that are placed in the same plane with an equally spaced gap between each other. The rotational-symmetric spiral channel consists of two countercoiled spirals that are vertically arranged in parallel. Then, the spirals are connected at the center and outer edge so that the spirals become a one-looped channel. As shown in the enlarged view of the channel connection, a MEMS piezoresistive cantilever is placed to block the flow in the channel. The cantilevers are placed at different positions in each spiral channel, one at the center edge and the other at the outer edge of the spiral channel, as shown in Figure 1(a-ii). The pair of piezoresistive cantilevers is connected to an amplifier circuit as a bridge circuit. When angular acceleration is applied to the sensor, the fluid inside the channel stays still due to inertia, while the channel itself rotates. Therefore, the inertial force is generated against the fluid rotationally shifting relative to the channel. At this moment, the inertial force causes differential pressure between the upper and lower surfaces of the cantilever because the cantilever blocks fluid flow. The differential pressure deforms the cantilever, as shown in Figure 1(a-iii). The deformation changes the resistance of the piezoresistor formed on the cantilever surface. The cantilevers of the rotational-symmetric spiral channels are used as a pair of the resistances of a bridge circuit in the two-gauge method. Then, the bridge circuit is connected to an amplifier circuit. The angular acceleration is calculated from the voltage change output via the amplifier circuit.

**Figure 2:**
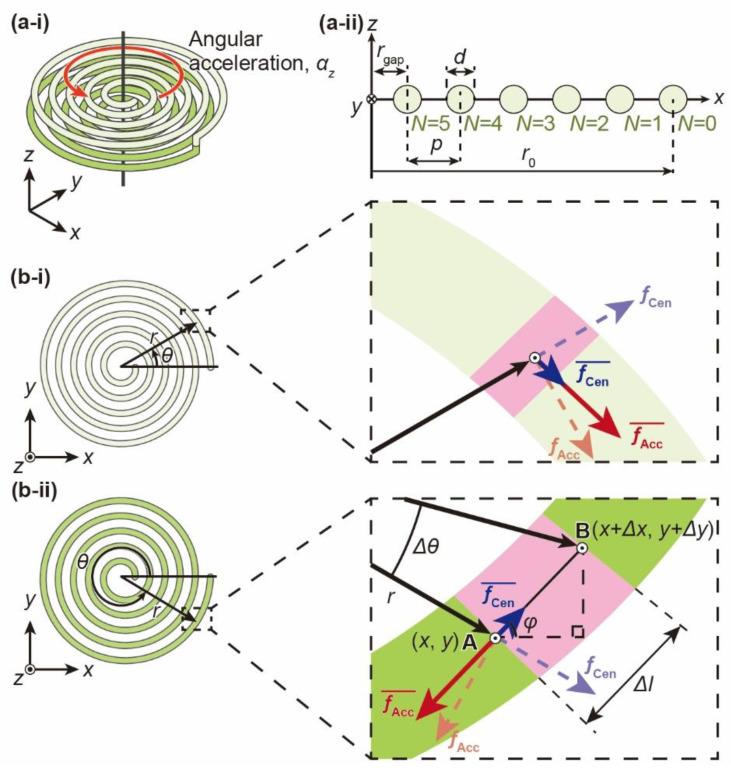
Illustration of the force applied to the fluid in the spiral channel. The dotted arrow shows the inertial force, *f*_Acc_, caused by the angular acceleration, and the centrifugal force, *f*_Cen_. The solid arrow shows the component force of the inertial force, $\bar{f_{\mathrm{Acc}}}$, and the centrifugal force, $\bar{f_{\mathrm{Cen}}}$ in the tangential direction to the channel. (**a-i**) Bird-eye view schematic image of the spiral channel and (**a-ii**) cross-sectional view and parameters of the upper spiral channel. The parameters are defined as follows: the number of turns, *N*, the outer radius, *r*_0_, the distance from the origin to the end of the spiral, *r*_gap_, the diameter of the channel, *d*, and the pitch of the spirals, *p*. (**b-i**) The upper spiral and an enlarged view and (**b-ii**) the lower spiral and an enlarged view.

Here, we focus on the theoretical differential pressure applied to the cantilever in detail. The differential pressure is generated from the circumferential directional force in the channel, whereas radial directional force does not affect the cantilever. If the channel is a simple circle, the inertial force due to angular acceleration is applied circumferentially, while the centrifugal force due to angular velocity is applied radially in the channel. Thus, only the inertial force affects the cantilever as the differential pressure. In the case of a spiral channel, the force direction is complicated. When the angular acceleration *α*_z_ around the *z*-axis is applied to the spiral channel, as shown in Figure 2(a-i), an inertial force *f*_Acc_ and a centrifugal force *f*_Cen_ are generated in the upper and lower spirals. The enlarged views in Figure 2(b-i) and Figure 2(b-ii) show the inertial force *f*_Acc_, the centrifugal force *f*_Cen_ and their components $\bar{f_{\mathrm{Acc}}}$ and $\bar{f_{\mathrm{Cen}}}$ in the circumferential direction. In the case of spiral channels, the centrifugal force *f*_Cen_ is not completely radial to the channel, so the differential pressure is affected by the centrifugal force *f*_Cen_ in addition to the inertial force *f*_Acc_. However, when the spirals, which are countercoiled to each other, are connected at the top and bottom, $\bar{f_{\mathrm{Acc}}}$ is doubled, while $\bar{f_{\mathrm{Cen}}}$ is canceled. This is because the force directions of $\bar{f_{\mathrm{Acc}}}$ and $\bar{f_{\mathrm{Cen}}}$ are the same and opposite at each countercoiled spiral, respectively. Thus, only the inertial force *f*_Acc_ affects the cantilever as the differential pressure even in the case of spiral channels.

The differential pressure caused by the angular acceleration is theoretically calculated. As shown in the enlarged view in Figure 2(b-ii), we focus on a small section of the spiral channel between point A, located at a rotation of *θ* from the beginning of the spiral, and point B, rotated by ∆*θ* from point A. The radius of the spiral channel at the small section is defined as *r*. The coordinates of each point are defined as (*x*, *y*), (*x*+∆*x*, *y*+∆*y*). (*r*, *θ*) corresponds to the polar coordinate of (*x*, *y*). The distance between points A and B is also defined as ∆*l*. The small section is oriented in the circumferential direction to the channel; the tilt angle *φ* to the *x*-axis is defined.

The inertial force *f*_Acc_ at the small section is calculated using the density of the fluid, *ρ*, the cross-sectional area of the spiral channel, *S*, the small section distance, ∆*l*, the radius of the channel at the small section, *r*(*θ*), and the applied angular acceleration, *α_z_*, as below.

(1) $$f_{\mathrm{Acc}}=-\rho \left(S\Delta l\right)r\left(\theta \right)\alpha_{z}$$

As mentioned above, only $\bar{f_{\mathrm{Acc}}}$ affects the differential pressure generated in the spiral. The differential pressure per unit volume, d*P_z_*, is calculated using the angle *φ*, the central angle *θ* and ∆*θ*, the small section distance ∆*l*, and the *x-* and *y*-axis displacements ∆*x* and ∆*y*. d*P*_z_ is expressed as:

(2) $$\begin{array}{l} dP_{z}=\bar{f_{\mathrm{Acc}}}/S\\ =\left[f_{\mathrm{Acc}}\sin\left(\varphi -\theta \right)\right]/S\\ =-\rho \Delta lr\left(\theta \right)\alpha_{z}\left(\frac{\Delta y}{\Delta l}\cos\theta -\frac{\Delta x}{\Delta l}\sin\theta \right)\\ =-\rho r{\left(\theta \right)}^{2}\alpha_{z}\Delta \theta \end{array}$$

Through the expansion of Equation (2) to the entire spiral channel, the entire differential pressure, ∆*P_z_*, is described as:

(3) $$\Delta P_{z}=2\int_{0}^{2N\pi }\left(-\rho r{\left(\theta \right)}^{2}\alpha_{z}\right)d\theta$$

where *N* is the number of turns of the spiral. Thus, the differential pressure, ∆*P_z_*, is obtained by substituting the spiral equation for *r*(*θ*) in Equation (3). Figure 2(a-ii) shows the parameters of the spiral channels used in this study. The spiral is a physical structure obtained by displacing the end of Archimedes’ spiral by *r*_gap_ from the origin. Even if the value of *r*_gap_ is changed, the spacing between the gaps in the nonspiral areas remains constant. Thus, rotational-symmetric spirals can be superimposed so that they fit into each other’s gaps. The number of turns, *N*, the outer radius, *r*_0_, the radius, *r*(*θ*), and the central angle, *θ*, of the spiral channels are described as:

(4) $$r\left(\theta \right)=r_{0}-\frac{\theta }{2N\pi }\left(r_{0}-r_{\mathrm{gap}}\right)\;\left(0\leq \theta \leq 2N\pi \right)$$

With the substitution of Equation (4) into Equation (3), ∆*P_z_* is calculated as:

(5) $$\Delta P_{z}=-\frac{4N\pi \rho \left(r_{0}{}^{2}+r_{0}r_{\mathrm{gap}}+r_{\mathrm{gap}}{}^{2}\right)\alpha_{z}}{3}$$

From Equation (5), it is confirmed that the differential pressure is proportional to the fluid density, the number of turns of the spiral, and approximately the square of the outer radius of the spiral. Similarly, the differential pressure, ∆*P*_ring_, across a simple ring channel of radius *r*_0_ is described as:

(6) $$\begin{array}{l} \Delta P_{\mathrm{ring}}=\int_{0}^{2\pi }\left(-\rho r_{0}{}^{2}\alpha_{z}\right)d\theta \\ =-2\pi \rho r_{0}{}^{2}\alpha_{z} \end{array}$$

Therefore, the ratio of the differential pressure between the spiral channel and the circular channel, *γ*, is calculated as:

(7) $$\gamma =\frac{\Delta P_{z}}{\Delta P_{\mathrm{ring}}}=\frac{2N}{3}\left(1+\frac{r_{\mathrm{gap}}}{r_{0}}+\frac{r_{\mathrm{gap}}{}^{2}}{r_{0}{}^{2}}\right)\approx \frac{2N}{3}$$

Compared to the simple ring channel, the spiral channel has a higher sensitivity because more channels can be lined up in the same area.

**Figure 3:**
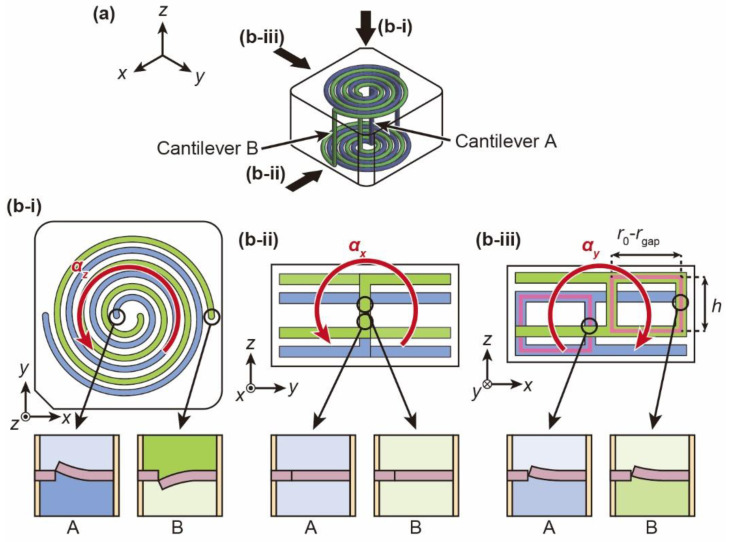
Schematic image of the cantilever deformation when triaxial angular acceleration is applied. (**a**) Bird-eye view of the spiral channel with each viewpoint. (**b**) The spiral channel and the deformation of the cantilever due to angular accelerations *α_z_*, *α_x_* and *α_y_* corresponding to the (**i**) *x*-*y* plane, (**ii**) *y*-*z* plane, and (**iii**) *x*-*z* plane, respectively.

The cancellation in the nontarget axis is also described theoretically. Figure 3a shows a schematic diagram of the sensor unit. The cantilevers are placed at the connections of the spirals at two locations: the center edge of the blue spiral channel and the outer edge of the green spiral channel. These cantilevers are designated cantilever A and cantilever B, respectively. Figure 3(b-i), Figure 3(b-ii) and Figure 3(b-iii) show the *x*-*y*, *y*-*z*, and *x*-*z* planes, respectively, when counterclockwise angular acceleration around the *x-*, *y-*, and *z*-axes is applied. In Figure 3(b-ii) and Figure 3(b-iii), the spiral channels are drawn vertically displaced for easier understanding because they overlap each other. When counterclockwise angular acceleration around the *z*-axis is applied to the sensor unit, clockwise inertial force with the same magnitude is generated in both channels because of the rotational symmetry of the two channels. Therefore, cantilevers A and B deform upward and downward, respectively. Then, the resistance changes in the two cantilevers are the same magnitude and opposite sign. The resistance changes are converted to the voltage change by using a pair of resistors as a bridge circuit in the two-gauge method. Thus, the voltage change becomes twice as large as that of a single cantilever. When angular acceleration around the *x*-axis is applied to the sensor unit, no differential pressure acts on the cantilevers because there is no circular flow path to generate inertial force. Thus, angular acceleration is not detected. When angular acceleration around the *y*-axis is applied to the sensor unit, clockwise inertial force is generated in both channels due to the circular portion of each channel. The differential pressure caused by the inertial force deforms both cantilevers A and B upward. The differential pressure ∆*P_y_* is calculated using the height of the spiral channel *h* and *r*_0_ and *r*_gap_ as below.

(8) $$\Delta P_{y}=2\rho h\left(r_{0}-r_{\mathrm{gap}}\right)\alpha_{y}$$

In this moment, the resistance changes are the same magnitude and the same sign, so they cancel each other. Thus, angular acceleration is not detected. Based on the above principles, only the angular acceleration around the *z*-axis is detected via the rotational-symmetric spiral channel.

## 3. Fabrication and Assembly

**Figure 4:**
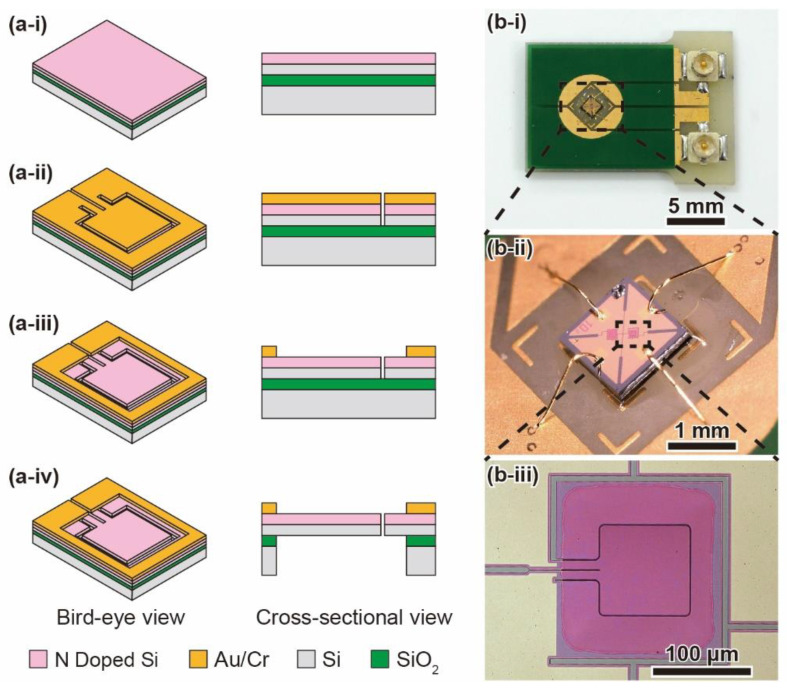
Fabrication process of the piezoresistive cantilever and photographs of the fabricated sensor chip and the cantilever. (**a-i**) Forming an N-type piezoresistive layer on the device Si layer of a silicon-on-insulator (SOI) wafer. (**a-ii**) Depositing a Au/Cr layer and etching the device Si layer. (**a-iii**) Patterning the Au/Cr layer. (**a-iv**) Etching the handle Si/SiO_2_ layers. Photographs of (**b-i**) the sensor element, (**b-ii**) the sensor chip, and (**b-iii**) the piezoresistive cantilever.

Figure 4a shows the fabrication procedure of the MEMS piezoresistive cantilever. The fabrication process is described in previous research [23]. The cantilevers are formed on a silicon-on-insulator (SOI) wafer. First, an N-type piezoresistive layer is formed on the device Si layer (Figure 4(a-i)). Next, a Au/Cr layer is formed on the device Si layer, and the cantilever shape is patterned. Then, the device Si layer is etched using inductively coupled plasma reactive ion etching (ICP-RIE) (Figure 4(a-ii)). Third, the Au/Cr layer is etched. (Figure 4(a-iii)). Finally, the handle Si layer and SiO_2_ layer are removed (Figure 4(a-iv)). The sensor chips are released from the wafer.

The sensor chip is 1.5 × 1.5 × 0.3 mm in size. The cantilever is 80 × 80 × 0.2 µm in size; the cantilever is significantly thin and easily deformed in the vertical direction but hardly deforms in the horizontal direction. The gap surrounding the cantilever is approximately 1 µm, which is so small that almost no air leaks out. Figure 4(b-i), Figure 4(b-ii), and Figure 4(b-iii) show photographs of the sensor element, the sensor chip, and the cantilever, respectively. The sensor chip is attached to a printed circuit board (PCB) at the position where a hole is located. On the sensor chip, there is a single cantilever and a dummy cantilever. The dummy cantilever does not change the resistance when differential pressure is applied because the handle Si layer is not etched.

**Figure 5:**
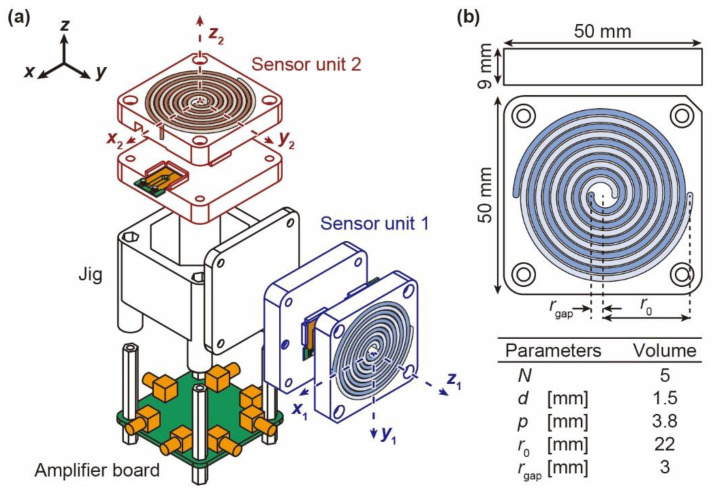
Proposed angular acceleration sensor consisting of two sensor units, an amplifier board, and a jig for connecting them. (**a**) Schematic illustration of the device assembly and the axis definition and (**b**) design of the sensor unit and parameter table of the spiral channel.

Figure 5a shows the device assembly diagram and the axis definition. The device consists of two sensor units, an amplifier board, and a jig to connect them. The housing of the two sensor units and the jig are fabricated using a stereolithography (SLA) 3D printer. We define the absolute coordinates of the device and the local coordinates of each sensor unit. The absolute coordinates are indicated by black arrows in Figure 5a. The sensor units attached to the *y*-axis and the *z*-axis are defined as sensor units 1 and 2, respectively. The local coordinate axes of sensor units 1 and 2 are defined as *x*_1_, *y*_1_, *z*_1_ and *x*_2_, *y*_2_, *z*_2_, respectively. They are drawn in the figure with blue and red dotted arrows. Each sensor unit is divided into upper and lower parts, and two sensor elements are inserted inside. The design of the sensor unit and the parameter table are shown in Figure 5b. The sizes of the upper and lower sensor units are both 50 × 50 × 9 mm. Hollow spiral channels are formed inside the sensor unit. To facilitate modeling with the 3D printer, the diameter of the spiral channels and the distance between the channels are designed to be 1.5 mm and 0.4 mm, respectively. Thus, the pitch of the spirals is set to 3.8 mm. Additionally, *r*_0_ and *r*_gap_ are set to 22 mm and 3 mm, respectively. The number of turns of the spiral is 5. This design maximizes the sensitivity while avoiding interference from O-rings that connect the sensor element to the sensor unit. From the design value of the spiral and Equations (5) and (8), the sensitivities of the single spiral channel for the *z*-axis and *y*-axis are calculated as follows.

(9) $$\begin{array}{c} \Delta P_{z}=1.4\times 10^{-2}\cdot \alpha_{z}\\ \Delta P_{y}=6.4\times 10^{-4}\cdot \alpha_{y} \end{array}$$

The sensitivity of the *z*-axis is approximately 20 times larger than that of the *y*-axis. The rotational-symmetric spiral channel is utilized, so the *z*-axis sensitivity is doubled and the *y*-axis sensitivity is canceled. Assuming that the cancellation reduces the sensitivity to 1/10, the ratio of the sensitivities of the *z*-axis and *y*-axis becomes approximately 400:1.

**Figure 6:**
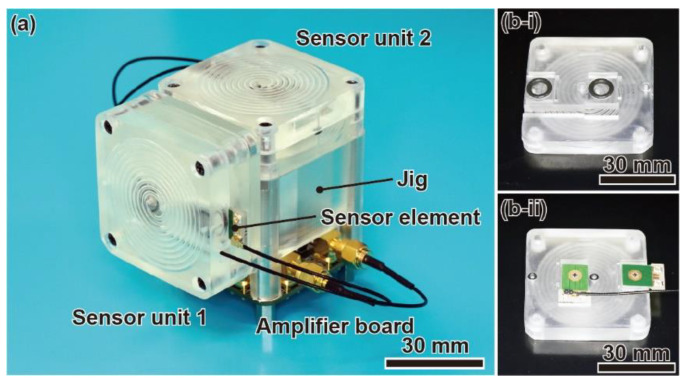
Photographs of (**a**) the developed angular acceleration sensor, (**b-i**) the upper part of sensor unit, and (**b-ii**) the lower part of sensor unit with sensor elements.

Figure 6a shows a photograph of the fabricated device. Spiral channels are formed inside the sensor units. In this study, we use air as the fluid to fill the spiral channels. The density and viscosity of air at room temperature of 293 K are as follows: density, *ρ* = 1.20 kg/m^3^, coefficient of viscosity, *μ* = 1.82 × 10^−5^ Pa·s. The cross-sectional area of the spiral channel is 1.5 × 1.5 mm^2^. To prevent air leakage in the spiral channels, O-rings are used at the connections of the sensor units, as shown in Figure 6(b-i) and Figure 6(b-ii).

## 4. Differential Pressure Calibration

**Figure 7:**
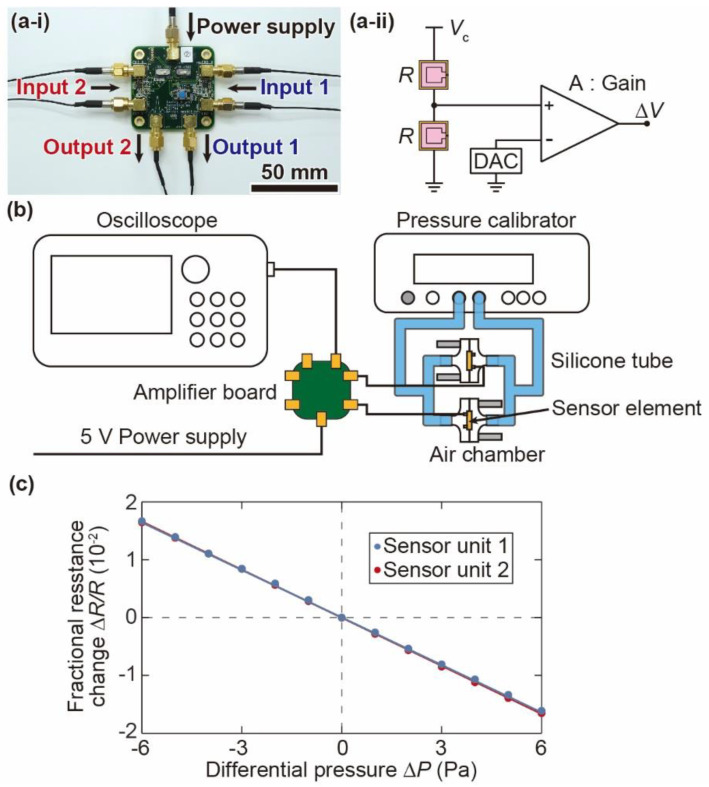
Differential pressure calibration of the sensor chip. (**a-i**) Photograph of the amplifier board used for the angular acceleration sensor. (**a-ii**) Schematic illustration of the bridge amplifier circuit. (**b**) Schematic image of the experimental setup, and (**c**) relationship between the applied differential pressure and the fractional resistance changes in the piezoresistive cantilevers.

Figure 7(a-i) and Figure 7(a-ii) show a photograph of the amplifier board used in the device and a schematic illustration of the bridge amplifier circuit, respectively. The amplifier board contains two sensor circuits, and two cantilevers of each sensor unit are connected to each circuit. The bridge circuit converts the resistance change of the piezoresistors into a voltage change. The voltage change, Δ*V*, is calculated using the initial resistance of the cantilever, *R*, the resistance change, Δ*R*, the gain of the instrumentation amplifier, *A*, and the bridge voltage *V*_c_ (*A* and *V*_c_ are set to 100 and 1 V) as below.

(10) $$\Delta V=\frac{\Delta R}{4R}\cdot A\cdot V_{c}=25\frac{\Delta R}{R}$$

We calibrate the sensor chips based on the differential pressure before assembling them into the sensor units. Figure 7b shows the experimental setup used for the differential pressure calibration. The two sensor elements are placed in different air chambers [24]. The chambers are connected to a pressure calibrator (Halstrup-Walcher GmbH, KAL 200, Kirchzarten, Germany) via silicone tubes. Then, static differential pressure is applied to the sensor elements from the pressure calibrator. We measure the output voltage of the amplifier using an oscilloscope (Yokogawa Electric Corporation, DL850, Musashino, Tokyo, Japan) while applying differential pressure from −6 Pa to +6 Pa with a 1 Pa interval to a pair of sensor elements. Using the obtained data and Equation (10), we calculate the fractional resistance change ∆*R*/*R* of two pairs of cantilevers at each differential pressure. Figure 7c shows the relationship between the differential pressure and the fractional resistance change. The fractional resistance changes of all cantilever pairs respond linearly to the differential pressure. The slope of the graph, ∆*R*/*R*/∆*P*, is defined as the sensitivity of the sensor unit for differential pressure. The sensitivities of sensor units 1 and 2 are calculated to be ∆*R*/*R*/∆*P* = 2.74 × 10^−3^/Pa and 2.77 × 10^−3^/Pa, respectively. In addition, the differential pressure resolution, which is calculated from the noise level ∆*R*_RMS_/*R*, is approximately 0.05 Pa. From the calibrated sensitivity for differential pressure and Equation (9), the sensitivity of the sensor unit to the angular acceleration for the target *z*-axis is expressed as follows:

(11) $$\Delta R/R/\ddot{\alpha_{z}}=3.9\times 10^{-5}/\left(\mathrm{rad}/s^{2}\right)$$

The sensitivity of the single spiral channel to the angular acceleration for the *y*-axis is expressed as follows with the calibrated sensitivity of one sensor chip and Equation (9).

(12) $$\Delta R/R/\ddot{\alpha_{y}}=0.1\times 10^{-5}/\left(\mathrm{rad}/s^{2}\right)$$

The ratio of sensitivities in Equations (11) and (12) is slightly lower than that of Equation (9). This difference is thought to be due to individual differences in the sensitivity of the sensor elements because the *z*-axis sensitivity is measured by two sensor elements, while the *y*-axis sensitivity is measured by a single sensor element.

## 5. Sensor Response

**Figure 8:**
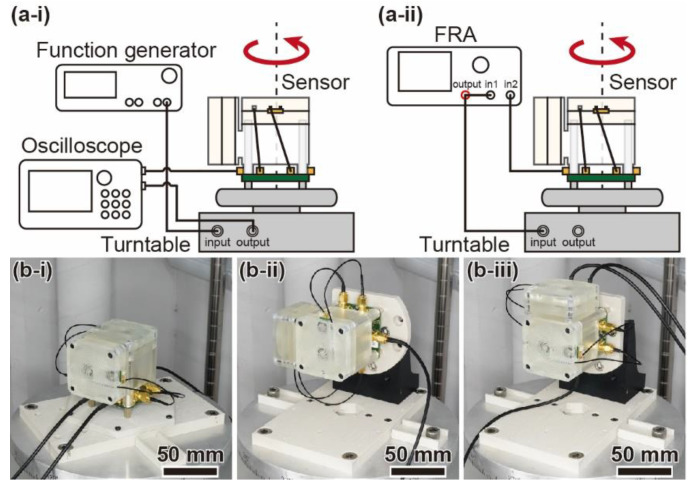
Measurement of the sensor response. (**a**) Schematic image of the experimental configurations to evaluate (**i**) the output waveforms and (**ii**) the frequency response to angular acceleration. (**b**) Photographs of the experimental setups when the angular acceleration around each axis is applied. (**i**) *z*-axis, (**ii**) *x*-axis, and (**iii**) *y*-axis.

First, we measure the output waveform to observe how the device responds to the input angular acceleration. Figure 8(a-i) shows a schematic diagram of the experimental setup. The angular acceleration sensor with the calibrated sensor elements is fixed on a turntable (Tamagawa Seiki Co., Ltd., TA4635N36, Iida, Nagano, Japan). With the input of a sinusoidal voltage signal to the turntable via a function generator (FG) (NF Co., WF1974, Yokohama, Kanagawa, Japan), the turntable is sinusoidally driven at a constant angular velocity, and angular acceleration is generated according to the angular velocity change. We measure the input and output waveforms when the turntable is driven by a sinusoidal wave. The amplitude and frequency of the driven angular velocity are 250°/s and 15 Hz, respectively. In the measurement, we apply a 500 Hz low-pass filter to reduce the noise generated by the turntable. Figure 8(b-i), Figure 8(b-ii), and Figure 8(b-iii) show photographs of the experimental setup with angular acceleration applied around the *z-*, *x-*, and *y*-axes (absolute coordinates), respectively.

**Figure 9:**
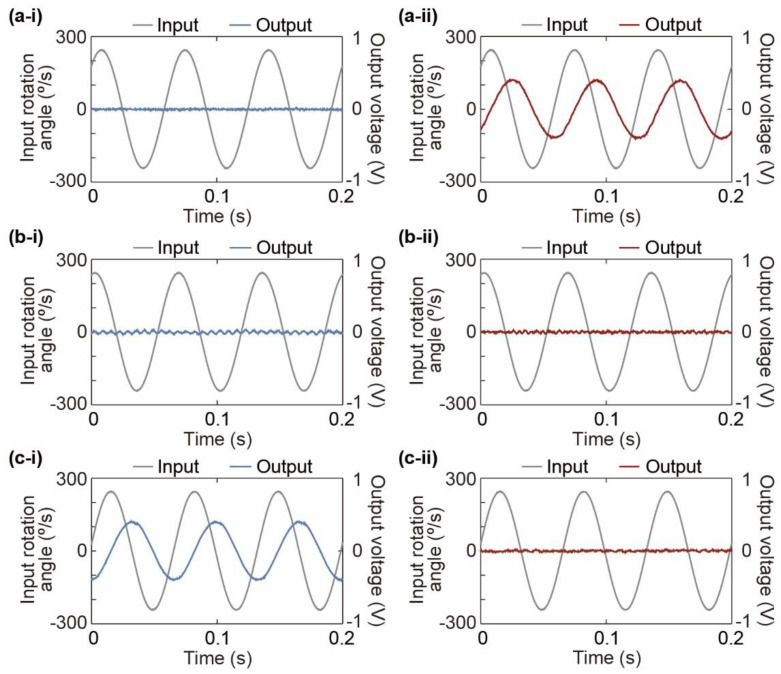
Input rotation angle of the turntable and output voltage of the sensor at 15 Hz. (**a**) *z*-axis direction of the proposed device, (**b**) *x*-axis direction of the proposed device, and (**c**) *y*-axis direction of the proposed device. (**i**) Sensor unit 1 and (**ii**) sensor unit 2.

Figure 9 shows the response waveforms of sensor units 1 and 2. From the experimental results, a sinusoidal wave of 15 Hz is generated for the *z*-axis direction of each sensor unit. The output waves delay the angular velocity of the input sinusoidal wave by π/2. On the other hand, there are no changes when the nontarget axial angular velocity is applied. In addition, there was a small output waveform of approximately 150 Hz, which is sufficiently larger than the input frequency of 15 Hz, as shown in Figure 9(b-i). The output is thought to be due to the mechanical rotational vibration of the experimental setup during the activation of the turntable. Therefore, each sensor unit responds to angular acceleration in the target axis direction and hardly responds to angular acceleration in the nontarget axis direction.

Second, we evaluate the frequency response using a frequency response analyzer (FRA) (NF Co., FRA5097, Yokohama, Kanagawa, Japan). Figure 8(a-ii) shows a schematic diagram of the experimental setup. A sinusoidal voltage, with an amplitude corresponding to 250°/s, is applied to the turntable from the FRA instead of the FG. Then, the sensor response is measured at each frequency as the frequency is varied from 1 to 15 Hz in 0.1 Hz increments.

**Figure 10:**
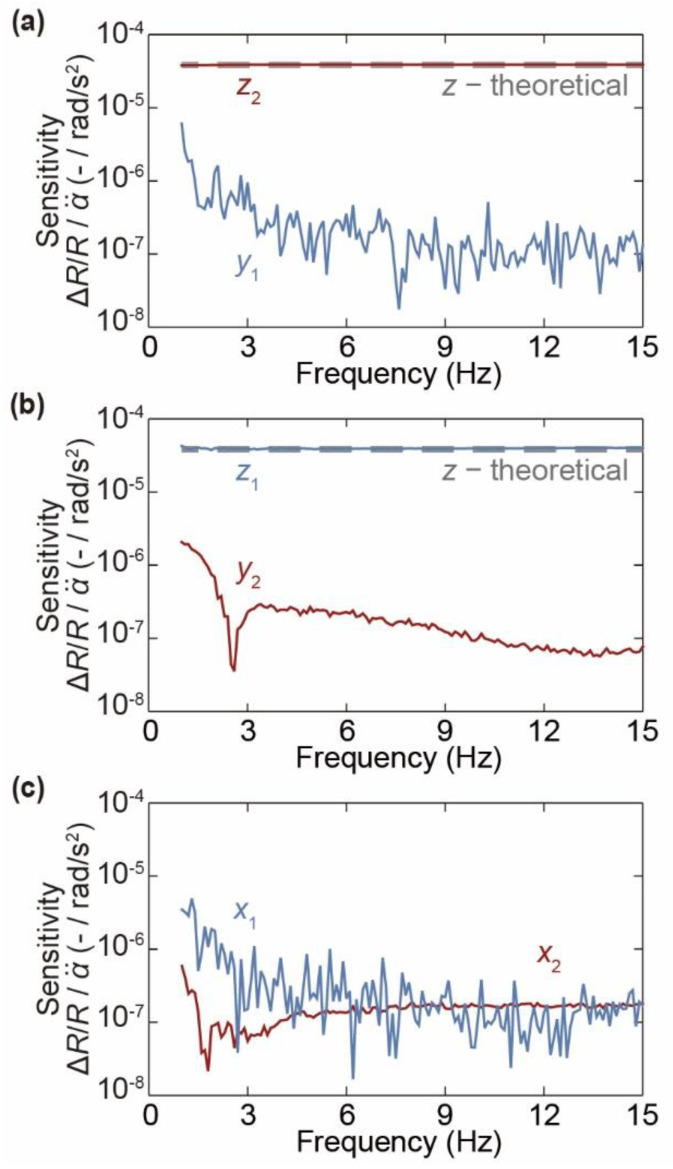
Relationship between the frequency of the applied angular acceleration and sensitivity for each axis of the sensor. (**a**) *z*-axis direction, (**b**) *y*-axis direction, and (**c**) *x*-axis direction.

Figure 10 shows the relationship between the frequency of the applied angular acceleration and the sensitivity for each axis of the sensor. From the experimental results, the target axis sensitivity is constantly 4.02 × 10^−5^/(rad/s^2^) for sensor unit 1 and 3.86 × 10^−5^/(rad/s^2^) for sensor unit 2. The differences from the theoretical value (Equation (11)) are just +3.1% for sensor unit 1 and −1.0% for sensor unit 2. Thus, the target axis sensitivity of each sensor unit is equal to the theoretical value. In addition, the nontarget axis sensitivity is approximately 1/10 to 1/20 of the target axis sensitivity in the low-frequency band. On the other hand, as the frequency increases, it becomes approximately 1/200 to 1/400. The nontarget axis sensitivity in the low-frequency band is mainly due to electrical noise because the applied angular acceleration is relatively low in the low-frequency band. Thus, the actual nontarget axis sensitivity is thought to be 1/200 to 1/400 of the target axis sensitivity. The target axis resolution was calculated as approximately 4 rad/s^2^ from the noise level of the cantilever and the sensor sensitivity.

**Figure 11:**
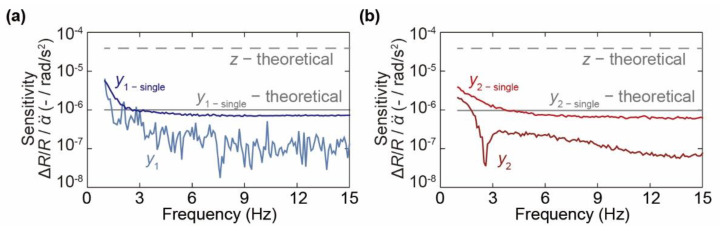
Canceling the *y*-axis sensitivity in each sensor unit. (**a**) Sensor unit 1 and (**b**) sensor unit 2.

Finally, we measure the response of a single spiral channel to angular acceleration around the *y*-axis to evaluate whether the sensor structure actually cancels out the *y*-axis sensitivity of each sensor unit. The cantilever and the dummy cantilever in one sensor element are connected to the amplifier circuit instead of two cantilevers of rotational-symmetric spiral channels. The dummy cantilever is not deformed by the differential pressure in the channel; thus, it compensates for the deforming cantilever. The sensitivity of each sensor unit in the *y*-axis direction is measured under the same experimental conditions as those in the previous experiments. As shown in Figure 11, the experimental results suggest that the *y*-axis sensitivity of each sensor unit responds at a value close to the theoretical value calculated in Equation (12). In addition, the *y*-axis sensitivity in the uncanceled state is approximately 1/40 of the target axis sensitivity, which is 10 times higher than the *y*-axis sensitivity canceled by using two sensor elements. Thus, it is suggested that the nontarget *y*-axis sensitivity is canceled by the rotational-symmetric sensor structure.

## 6. Response to Biaxial Angular Acceleration

**Figure 12:**
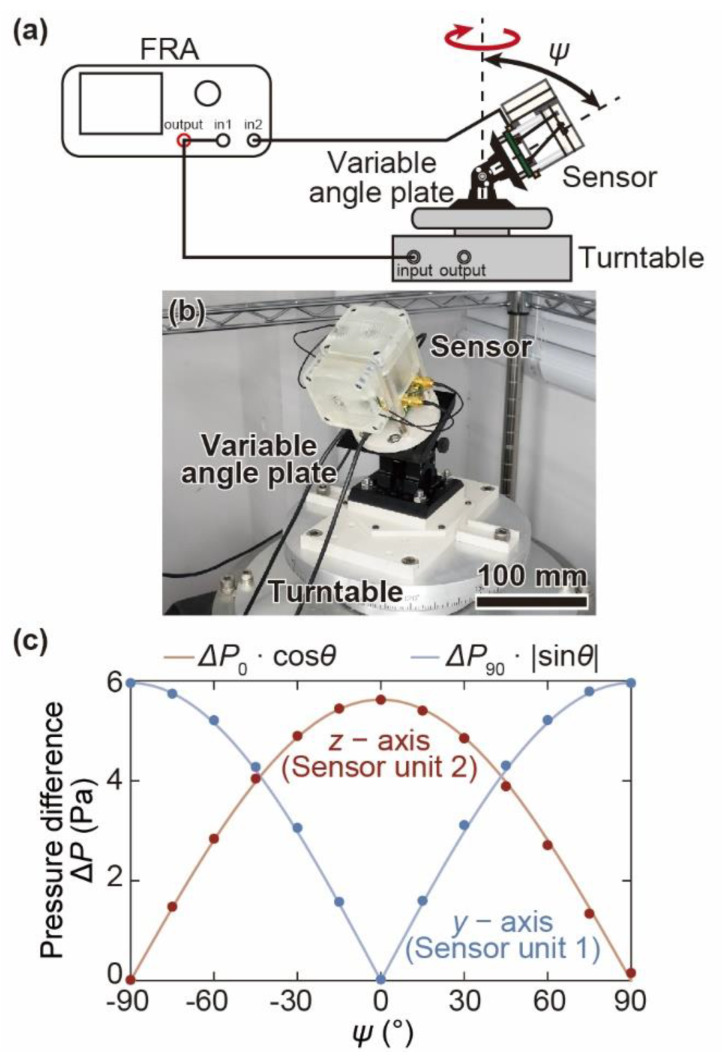
Measurement of biaxial angular acceleration. (**a**) Schematic image of the experimental setups for measuring the response of the sensor to angle, (**b**) photographs of the experimental setups when *ψ* = 30°, and (**c**) the relationship between the angle of the device and the differential pressure of the target axis. Each curve is a sinusoidal curve multiplied by the differential pressure applied to the cantilever by the angular acceleration around the target axis.

The sensor response to biaxial angular acceleration is evaluated. Figure 12a shows a schematic diagram of the experimental setup. The angular acceleration sensor is fixed on the turntable with a tilt angle with a variable-angle plate (Thorlabs, Inc., AP180/M, Newton, NJ, USA). The turntable is driven by a sinusoidal wave with an amplitude of 250°/s at 15 Hz, the same as in previous experiments. The sensor response in the absolute coor dinate *z*-axis and *y*-axis directions, which corresponds to the response of the target axis of sensor units 1 and 2, is measured as *ψ* and is changed from −90° to +90° in 15° increments. For example, Figure 12b shows a photograph of the experimental setup with the sensor tilted 30°. Figure 12c shows the relationship between the tilt angle and the differential pressures ∆*P*, which is defined as the amplitude of the fitted sinusoidal curve of each measured sensor output of sensor units 1 and 2. The plotted points are the measured data from when the experiment is conducted at each angle. Each curve is a sinusoidal curve multiplied by the differential pressure, as angular acceleration is applied to the target axis in each sensor unit. The magnitudes of the differential pressure follow a sinusoidal curve. Additionally, the phase of the magnitudes shifts 90°.

The experimental results indicate that each axis sensor unit detects the angular acceleration only of one axis and shows little reaction to the angular accelerations of the other axes. It is confirmed that the sensor is able to independently detect the biaxial components of angular acceleration without crosstalk from the other axis’ component. The experimental results also suggest that our proposed sensor requires no complicated calculations to extract one axial component of angular acceleration. This advantage facilitates the actual use of MEMS sensors.

## 7. Conclusions

In this study, we fabricated and evaluated a biaxial angular acceleration sensor using MEMS cantilever elements and rotational-symmetric spiral channels. The proposed device consisted of two orthogonal sensor units and an amplifier board, and angular acceleration in two axes was independently detectable because each sensor unit realized uniaxial sensitivity with low crosstalk. Each sensor unit consisted of rotationally symmetric spiral channels and cantilevers, and the cantilevers detected the inertial force generated in the spiral channels with high sensitivity. The parameters of the spiral channel included a diameter of 1.5 mm, a maximum radius of 22 mm, and 5 turns. The relationship between the target axial angular acceleration and the differential pressure generated was expressed as ∆*P_z_* = 1.4 × 10^−2^∙*α*_z_. The spiral channel was arranged in the rotation-symmetric position, and the target axis angular acceleration was detected by subtracting the output of the two cantilevers via a bridge circuit, while the nontarget axis angular acceleration was canceled.

In the experimental results, the sensor response was constant for angular acceleration from 1 to 15 Hz around the target axis. Additionally, the sensitivities were 4.02 × 10^−5^/(rad/s^2^) for sensor unit 1 and 3.86 × 10^−5^/(rad/s^2^) for sensor unit 2, which were similar to the theoretical value. On the other hand, the nontarget axis sensitivity was canceled by the rotation-symmetric sensor structure, and the response was approximately 1/400 of the target axis. When the angular acceleration sensor was tilted, the magnitudes of the generated differential pressure followed a sinusoidal curve according to the tilt angle. It was confirmed that the target axis sensitivity was not affected by the sensitivity of other axes.

In this study, we focused on the biaxial detection of angular acceleration; thus, the experiments were conducted in the frequency range from 1 to 15 Hz. However, since we were previously able to apply the same detection principle to the bandwidth from 0.1 to 100 Hz, the bandwidth of the sensor was the same or larger [21]. The target axis resolution was calculated as approximately 4 rad/s^2^ from the noise level of the cantilever and the sensor sensitivity. The resolution can be enhanced to approximately 1/1000 by altering a fluid such as silicone oil to fill the channel. This resolution is larger than those of previous sensors with similar sensor sizes [16,19,22]. Thus, the biaxial angular acceleration components were simply calculated from the corresponding outputs of the two sensor units with no complicated calculations. As described above, the proposed sensor structure will be useful for biaxial angular acceleration sensors with low crosstalk. Furthermore, the proposed sensor can be realized as a triaxial angular acceleration sensor by adding another sensor unit that is orthogonal to the two sensor units. Such sensors are expected to be useful for monitoring the rotational vibration of mobile objects such as robots.

## Data Availability

The data presented in this study are available on request from the corresponding author.
